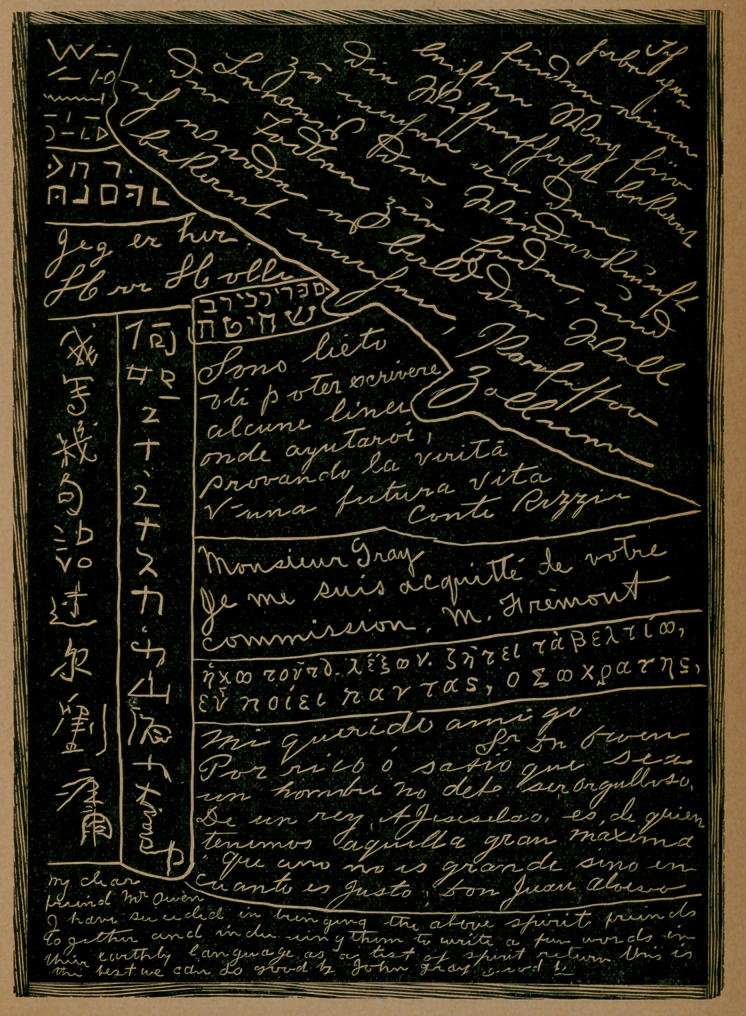# The Occult Forces

**Published:** 1887-04

**Authors:** 


HALL'S
JOURNAL OF HEALTH

TRUTH DEMANDS NO SACRIFICE; ERROR CAN MAKE NONE.

VOL. 34. APRIL, 1887. No. 4.

### THE OCCULT FORCES.

"'Tis Heaven itself that points out an hereafter,
And intimates eternity to man."

ADDISON.

We have heretofore taken occasion to lay before our readers certain well authenticated phenomena, which can only be rationally accounted for upon the hypothesis that the psychical or soul element in man is capable of expressing itself in various ways in a manner independent of his physical organism. In presenting these evidences, our object has been, more than all, to counteract a growing tendency toward *materialism* among a class of independent thinkers, who find in nature so many essential contradictions of the unphilosophical teachings of the different orders of theologic schoolmen, who are too apt to sacrifice the *spirit* to the *letter* of their texts, as gleaned from the chronicles of unlettered and semi-barbarous races, to be ecclesiastically upheld, as *per se* too sacred to be questioned, even when at variance with nature's divine revelations, which measure the periods of time, and mark the progress of the inhabitable globe toward the accomplishment of its destiny, as the dwelling place of intelligent beings, at all times subject to the rule and order which pervade the universe of matter, from the invisible atom to the innumerable spheres which accomplish their everlasting rounds in the infinitude of space.

Strictly considered, there never was and never can be, in the unappreciable realm of nature, either *accident* or *miracle.* All is in accordance with undeviating law, which to transgress, even in the slightest degree, is to suffer accordingly. In the ages which succeeded the destruction of nearly all that had been gained to science and the knowledge of things celestial, it was the vulgar habit to ascribe to *magic* everything that transcended the popular understanding, as it is in our day to hold as *miracu-*

*lous*, or independent of law, many of the happenings which cannot be explained by understood principles.

This misconception has given rise to a belief in *special providences*, as applied to events, to races and even to individuals, as if the Almighty Ruler of millions and millions of worlds, compared with which our own is a mere atom in a universe of atoms, would swerve from His eternal principles to invidiously shield or chastise a single element so inferior as man in the order of creation.

What importance must one arrogate to himself to be able to assign to the Creator an office so flattering to his vanity and selfishness!

In dealing with facts and the evidences of facts, so far removed from those which, from their frequency, have familiarized themselves with the common experiences of life as to elude investigation by comparison or agreement with them, our sole purpose has been to make man better acquainted with himself, that by his own unfoldment he may be brought to an understanding of those subtile laws of being which reach beyond the merely physical, into the almost unknown and unexplored realm of the psychological or spiritual.

It has never been our purpose to antagonize any theological system or to incite contention between their different orders, under whatever name, or indulging in whatever ceremonial forms. With such matters we have nothing to do. But with such as relate to man, his integral being, entity, we have everything to do, nor shall we falter in our duty out of any worldly consideration.

In previous numbers of the JOURNAL it has been our endeavor to show that man is possessed of what has been termed a double consciousness ; one dependent upon his physical machinism for sensation, and the other depending solely upon his psychological perceptions. In pursuance of our resolution, we now lay before our readers a more difficult problem, and shall content ourselves with giving the simple facts as they occurred, leaving it to their own perceptions to make any solution of them that will satisfy their individual minds. It is well known that there are certain delicately organized individuals in whose presence, under favorable cir-ditions, a class of phenomena occur which are in no wise assisted by human endeavor, beyond a passive submission to be used as an instru-mentality of force, in which the intellectual faculties of the psychic have no share and are frequently held in enforced suspension by a state of trance.

The *fac-simili* slate-writing, which illustrates this article, in which no change has been made beyond its reduction in size, was obtained under the following circumstances: In December last, Mr. John J. Owen, editor of the *Golden Gate*, San Francisco, Cal., after some preliminary arrangements had been complied with, repaired with Mrs. Owen to the rooms of Mr. Evans, a well-known psychic of that city, for a sitting with him, as it is termed. Having placed his private mark upon an ordinary framed slate, with blank surfaces, a bit of slate pencil was placed upon the plain wooden surface of a table, and the slate laid over it, with only the space caused by the frame projection between the lower surface of the slate and the upper surface of the table. Mr. Evans, sitting opposite to Mr. and Mrs. Owen, then touched the outer edge of the slate, which was being held under their open palms.

In a space of time not exceeding six minutes, the intimation was given that the object of their visit had been attained, and, on raising the slate, its entire lower surface was found to be covered with writings, in different hands, different chirographical modes, and no less than *eleven different languages*, which it is safe to assert no one person on this continent is master of. The following is as nearly a literal translation of the foreign languages, ancient and modern, as their English rendering will admit of :

GERMAN—I have found an easy way for making known to science the proof of the return of the dead to this earth, and I shall soon give it to the world.

PROFESSOR ZOLLNER.

ITALIAN—I am glad to be able to write you a few lines to aid in proving the truth of a future life. COUNT ROZZIA.

FRENCH—*Monsieur Gray :* I have acquitted myself of your commission.

M. FREMONT.

GREEK—I come to say this—seek for better things—think well of all.

SOCRATES.

SPANISH—*My Dear Friend, Sr. Don Owen :* Rich or wise as a man may be, don't let him be proud. It is from a King, Agesilaus, we have that grand maxim "that one is not great only as far as he is just." DON JUAN ALVISO.

NORWEGIAN—I am here. HERR HOLLE.

CHINESE—I write a few words to you. LU YEUN.

JAPANESE—How do you do ? OYAMA GENTURA.

HEBREW—(This is a name of a book describing the killing of animals according to the Jewish rites.)

EGYPTIAN—Yea, the spirit of man shall live forever.

OLD ASIATIC—Tom Paine (written in the Assyrian cuniform characters).

We give these facts precisely as we have received them, with the assurance that they may be *relied upon as true.* Had we not had similar experiences ourself, covering a long period of patient investigation, we should not be so ready to venture the assertion.

Of one thing we feel assured, and that is that these phenomena completely vanquish the theory of their production by any process of legerdemain or mind reading, for Mr. Owen says, in reference to them, that with the exception of a little knowledge of French and Spanish, the English is the only language with which he or his wife is familiar, and there were present at the sitting only Mr. Evans and themselves.

As to the materialist, we lay before him these sublime evidences which point unerringly toward a continued conscious existence in the hope that through their teaching he may be induced to turn his thoughts upward, till he shall be able to realize for man a destiny higher than that of

"A poor player,
That struts and frets his hour upon the stage,
And then is heard no more."

## Figures and Tables

**Figure f1:**